# Progress of *Journal of Functional Morphology and Kinesiology* in 2021

**DOI:** 10.3390/jfmk7010024

**Published:** 2022-02-23

**Authors:** Giuseppe Musumeci

**Affiliations:** 1 Department of Biomedical and Biotechnological Sciences, Anatomy, Histology and Movement Sciences Section, School of Medicine, University of Catania, Via S. Sofia 87, 95123 Catania, Italy; g.musumeci@unict.it; 2 Research Center on Motor Activities (CRAM), University of Catania, 95123 Catania, Italy; 3 Department of Biology, Sbarro Institute for Cancer Research and Molecular Medicine, College of Science and Technology, Temple University, Philadelphia, PA 19122, USA

## 1. Looking Back on 2021

The *Journal of Functional Morphology and Kinesiology* (*JFMK*, ISSN: 2411-5142), which was first released in March 2016, has gone from strength to strength in 2021. This journal provides a forum for research studies on functional morphology and kinesiology and the regulatory functions of movement. *JFMK* meets the growing demand for high-quality, peer-reviewed international journals. As an open access journal, *JFMK* has the benefits of being accessible to all readers and having high visibility, and supplies Digital Object Identifier (DOI), ORCID, and CrossRef information to all researchers. We are indexed in Scopus (Elsevier’s abstract and citation database), PubMed, PMC, DOAJ (Directory of Open Access Journals), Scilit (a comprehensive, open access scholarly database, developed and maintained by MDPI), Google Scholar, World Health Organization Hinari, FSTA—Food Science and Technology Abstracts (IFIS) and the Norwegian Register for Scientific Journals, Series and Publishers (NSD). Our full texts are archived in CLOCKSS (Digital Archive), e-Helvetica (Swiss National Library Digital Archive), and J-Gate (Informatics India). 

In 2021, *JFMK* was covered by SCImago Journal Rank with a 1.7 Citescore, reaching Q2 in the following research fields: Anatomy, Histology, Orthopedics and Sports Medicine, Physical Therapy, Sports Therapy, Rehabilitation and Rheumatology.

The CiteScoreTracker 2021 score of our journal in Scopus in 2021 was 2.7, higher than the previous year, demonstrating the growth in citations of our journal. We hope to be included in Web of Science in the near future. 

*JFMK* is a member of the Committee on Publication Ethics (COPE). To verify the originality of content submitted to our journals, we use iThenticate to check submissions against previous publications. MDPI works with Publons to provide reviewers with credit for their work. In addition, MDPI Scitations Alert lets authors know about new publications in their research field. 

The journal publishes articles focusing on molecular, cellular, tissue, system and the whole body responses to a broad range of physical activities. Furthermore, the journal provides a forum for analysis of the structure, function, development, and evolution of the cells and tissues of the musculoskeletal system and associated clinical disorders. Thanks to continuous support from our Editorial Board, authors, reviewers, and editorial staff, the *Journal of Functional Morphology and Kinesiology* has experienced a fruitful year [1,2,3,4], as demonstrated in our statistics https://www.mdpi.com/journal/jfmk/stats, accessed on 14 December 2021. 

Indeed, the number of published manuscripts has increased from 88 in its 2020 volume to 98 in 2021. We rejected 29.30% of contributions to ensure publications in our journal remained of high quality. The *Journal of Functional Morphology and Kinesiology* receives more manuscripts than it is able to publish, and the decision as to which papers are accepted or rejected is a difficult one. This decision is based on several factors, including originality, experimental design, scientific quality, data interpretation, clarity, and the quality of the written English, in order to continually to maintain the high standards of our journal.

In 2021, various Special Issues were organized and set up thanks to the huge support of our editors. They include the following: “Exercise Evaluation and Prescription-2nd Edition”, edited by Prof. Dr. Cristina Cortis, Dr. Andrea Fusco and Prof. Dr. Carl Foster [5]; “Role of Exercises in Musculoskeletal Disorders-4th Edition”, edited by Prof. Dr. Giuseppe Musumeci and Dr. Silvia Ravalli [6]; “Fractures Management in Upper and Lower Limbs”, edited by Prof. Dr. Vito Pavone [7]; “Health Promotion in Children and Adolescents through Sport and Physical Activities-3nd Edition", edited by Prof. Dr. Antonino Bianco [8]; “Working Group in Sports Medicine”, edited by Prof. Dr. Eleftherios Kellis, Prof. Dr. Vito Pavone, Prof. Dr. Giuseppe Musumeci, Dr. Gianluca Vadalà and Prof. Dr. Peter Hofmann [9]. “Exercise and Neurodegenerative Disease 2.0”, edited by Dr. Grazia Maugeri and Prof. Dr. Velia D’Agata [10]; “Psychology of Development and Education Applied to Movement 2022”, edited by Prof. Dr. Marianna Alesi [11]; “Strength Training for Human Health and Performance”, edited by Dr. James Fisher, Dr. Patroklos Androulakis-Korakakis and Dr. Milo Wolf [12]; “The Challenges of Open Water Swimmers”, edited by Prof. Dr. Maria Francesca Piacentini and Dr. Veronica Vleck [13]; “The Reverse Shoulder Arthroplasty”, edited by Dr. Kotaro Yamakado [14]; “Strength and Conditioning for Team Sports”, edited by Dr. Filipe Manuel Clemente [15]; “Influence of Motivational and Preferred Music on Performance in Sport and Exercise”, edited by Dr. Christopher Ballmann [16]; “Exercise Training and Diabetes Mellitus”, edited by Dr. Giorgio Orlando, Dr. Jonida Haxhi and Dr. Luciana Labanca [17]; “New Advances in Human Posture and Movement 2021”, edited by Prof. Dr. Olivier Hue [18]. “Kinesiotaping in Sport and Rehabilitation Settings”, edited by Dr. Diego Minciacchi, Dr. Riccardo Bravi and Dr. Erez James Cohen [19]. “Motor Competence, Physical Activity and Health”, edited by Prof. Dr. Vítor P. Lopes and Prof. Dr. Luis Paulo Rodrigues [20]. “Overweight and Exercise Nutrition”, edited by Dr. Felix Strollo [21]. “Muscle Strength and Power”, edited by Prof. Dr. Jeffrey M. McBride and Dr. Jared Skinner [22]. “Movement Analysis 2.0”, edited by Dr. Luís Silva [23]. “Exercise Evaluation and Prescription-3rd Edition”, edited by Prof. Dr. Cristina Cortis, Dr. Andrea Fusco and Prof. Dr. Carl Foster [24].

In 2021, seven distinguished scientists joined the Editorial Board: Dr. Gerwin Alexander Bernhardt (Department of Orthopedics and Trauma, Medical University Graz, Graz, Austria); Prof. Dr. Carl Foster (Department of Exercise and Sport Science, University of Wisconsin-La Crosse, La Crosse, WI, USA); Dr. Felix Strollo (IRCCS San Raffaele Pisana, Rome, Italy); Prof. Dr. Vítor P. Lopes (Escola Superior de Educação de Lisboa, Instituto Politécnico de Lisboa, 1549-003 Lisboa, Portugal); Dr. Diego Minciacchi (Department of Experimental and Clinical Medicine, University of Florence, Florence, Italy); Dr. Steven J. Coles (School of Science and the Environment, University of Worcester, Worcester, UK); Prof. Dr. Tibor Hortobagyi (University Medical Center Groningen, University of Groningen, 9713 AV Groningen, The Netherlands), reaching a total of 81 Editorial Board members, 7 Advisory Board members, and the Editor-in-Chief.

All articles published in the *Journal of Functional Morphology and Kinesiology* are published in full open access format. In order to provide free, unlimited access to readers and to cover the costs of peer-review, copyediting, typesetting, long-term archiving, and journal management, an article processing charge (APC) of CHF 1600 (Swiss Francs) applies to papers that are accepted after peer-review.

## 2. Looking Forward to 2022

In 2022, we shall continue our efforts to improve the journal through further growth and increased visibility. 

In order to achieve this target and lay a strong foundation for publications in 2022 and application for indexing, we have made the following plans:

- Follow up planned papers from Editorial Board members;
- Contact international conferences recommended by the Editor-in-Chief or by Editorial Board members and establish media partnerships with them to increase the visibility of *JFMK* among scholars;
- Communicate with Editorial Board members more frequently and seek their input and expertise for journal development;
- Share high-quality papers through social media (e.g., LinkedIn, Twitter, and Facebook) and increase online readership;
- Reduce the processing time of each submitted manuscript;
- Have publications indexed by the Emerging Sources Citation Index (Web of Science), EMBASE (Elsevier) and Web of Science—Clarivate;
- Improve Citescore in the SCImago Journal Rank for kinesiology-related sections such as Anatomy, Histology, Orthopedics and Sports Medicine, Physical Therapy, Sports Therapy, Rehabilitation and Rheumatology;
- Achieve our first Impact Factor released by the Clarivate Analytics;
- Host the *JFMK* Best Paper Award and the *JFMK* Travel Grant Award for our authors;
- Garner support from sponsors so our editors can participate in international conferences and disseminate our journal.

Since 2021, MDPI has included the Academic Editor’s name on published articles, where they have accepted that manuscript after full peer-review. This supports greater transparency for the readership, demonstrates the care that our Academic Editors take in making decisions, and offers full acknowledgement of the effort put in when making expert judgements about the suitability of a manuscript for publication. We strongly believe that this will also support the rigorous and robust quality of our peer-review process.

We hope that you share our enthusiasm for this journal, and we look forward to working with you to make *JFMK* a leader in its field. Your contributions are vital for the success of this new journal. We look forward to receiving your contributions (papers, reviews, etc.), and proposals for Special Issues are always welcome.

I have personally found this to be quite a challenge, not helped by COVID, but more than that, due to the special position that *JFMK* is trying to navigate from in the highly competitive publishing landscape. I wish you a healthy and prosperous new year and look forward to continuing to expand the reach and impact of the journal with your help and support.

I will also take this opportunity to warmly thank the following for their confidence: our authors, readers, and reviewers, as well as our editorial advisors, eminent scientists in these fields that, with their experience and important suggestions, guide us in this great enterprise; our excellent Editorial Board members whose depth of experience covers a very broad spectrum of different disciplines related to the morphology and kinesiology arenas; the managing editor Ms. Molly Lu for her tremendous support, the publishing manager Dr. Peter Ribar, and the other members of the Editorial office that, day after day, thanks to their valuable contributions, ensure the growth of this journal; and, finally, all members of our teams in Basel, Barcelona, Beijing, Belgrade, Romania, Tokyo, and Wuhan, as well as our sponsors.
